# Caspofungin induces the release of Ca^2+^ ions from internal stores by activating ryanodine receptor-dependent pathways in human tracheal epithelial cells

**DOI:** 10.1038/s41598-020-68626-7

**Published:** 2020-07-16

**Authors:** Sabrina Müller, Christian Koch, Sebastian Weiterer, Markus A. Weigand, Michael Sander, Michael Henrich

**Affiliations:** 1 Department of Anesthesiology and Intensive Care Medicine, University Hospital of Giessen and Marburg, Giessen, Germany; 2 Department of Anesthesiology and Intensive Care Medicine, Heidelberg University Hospital, Heidelberg, Germany; 3 Department of Anesthesiology and Intensive Care Medicine, Vidia Clinic Karlsruhe St. Vincentius gAG, Karlsruhe, Germany

**Keywords:** Fungal infection, Medical research, Pathogenesis

## Abstract

The antimycotic drug caspofungin is known to alter the cell function of cardiomyocytes and the cilia-bearing cells of the tracheal epithelium. The objective of this study was to investigate the homeostasis of intracellular Ca^2+^ concentration ([Ca^2+^]_i_) after exposure to caspofungin in isolated human tracheal epithelial cells. The [Ca^2+^]_i_ was measured using the ratiometric fluoroprobe FURA-2 AM. We recorded two groups of epithelial cells with distinct responses to caspofungin exposure, which demonstrated either a rapid transient rise in [Ca^2+^]_i_ or a sustained elevation of [Ca^2+^]_i_. Both patterns of Ca^2+^ kinetics were still observed when an influx of transmembraneous Ca^2+^ ions was pharmacologically inhibited. Furthermore, in extracellular buffer solutions without Ca^2+^ ions, caspofungin exposure still evoked this characteristic rise in [Ca^2+^]_i_. To shed light on the origin of the Ca^2+^ ions responsible for the elevation in [Ca^2+^]_i_ we investigated the possible intracellular storage of Ca^2+^ ions. The depletion of mitochondrial Ca^2+^ stores using 25 µM 2,4-dinitrophenol (DNP) did not prevent the caspofungin-induced rise in [Ca^2+^]_i_, which was rapid and transient. However, the application of caffeine (30 mM) to discharge Ca^2+^ ions that were presumably stored in the endoplasmic reticulum (ER) prior to caspofungin exposure completely inhibited the caspofungin-induced changes in [Ca^2+^]_i_ levels. When the ER-bound IP_3_ receptors were blocked by 2-APB (40 µM), we observed a delayed transient rise in [Ca^2+^]_i_ as a response to the caspofungin. Inhibition of the ryanodine receptors (RyR) using 40 µM ryanodine completely prevented the caspofungin-induced elevation of [Ca^2+^]_i_. In summary, caspofungin has been shown to trigger an increase in [Ca^2+^]_i_ independent from extracellular Ca^2+^ ions by liberating the Ca^2+^ ions stored in the ER, mainly via a RyR pathway.

## Introduction

The mucociliary clearance of lower airways contributes to the removal of debris and pathogens that are trapped in the upper mucus layer from the lower airways. This complex mechanism is driven by cilia-bearing cells of the bronchiolar and tracheal epithelium. The kinocilia of these cells bear over 200 motor proteins as a propelling apparatus, including dynein, the ATP hydrolyzing enzyme^1,2^. Under basal conditions, cilia beat continuously without external stimulation but can beat faster when necessary. This elevated beating frequency depends on several interdepending signal transduction cascades, including changes in intracellular concentrations of Ca^2+^ ions ([Ca^2+^]_i_)^3–6^. However, most signal cascades in these cells depend on at least a transient [Ca^2+^]_i_ to be activated in order to allow the cilia to beat faster for prolonged periods^7,8^. Thus, the regulation of [Ca^2+^]_i_ is a cornerstone for many cellular signal cascades and cell functions. In epithelial cells in the airway, different canonical Ca^2+^ stores or Ca^2+^ influx pathways are involved in the regulation of [Ca^2+^]_i_. The known intracellular stores are the endoplasmic reticulum (ER) and the mitochondria. The ER can reduce [Ca^2+^]_i_ via activation of the sarcoplasmic ATPase (SERCA)-carrying Ca^2+^ ions into the ER. The release of Ca^2+^ ions from the ER is mainly orchestrated by activating the ryanodine receptors (RyR) or IP_3_ receptors to enhance [Ca^2+^]_i_. There may also be alternative pathways that lead to Ca^2+^ leakage from the ER; however, these are still unknown. Mitochondria can buffer Ca^2+^ ions when [Ca^2+^]_i_ exceeds a threshold of 500 nM and they slowly release Ca^2+^ into the cytosol when [Ca^2+^]_i_ falls below the aforementioned threshold^9^. It is still not known whether further intracellular Ca^2+^ stores such as lysosomes contribute to the regulation of [Ca^2+^]_i_.

Different plasma membrane-bound Ca^2+^ channels are known as Ca^2+^ influx pathways, which include transient receptor potential (TRP) channels, store-operated calcium (SOC) channels or voltage-dependent calcium channels that can all enhance [Ca^2+^]_i_. Consequently, any disturbance or rise in [Ca^2+^]_i_ in epithelial airway cells can be achieved by the activation of different Ca^2+^ pathways or by liberating them from internal stores. Elevated [Ca^2+^]_i_ directly contribute to altered cell function, e.g., mucociliary clearance or mucus secretion, which can either lead to colonization of the lower airways by pathogens or can lead to improved clearance rates. When treating mycotic infections in critically ill patients, echinocandines represent the established first-line therapy of antimycotic substances including caspofungin. Caspofungin is used under clinical conditions, with a high capability to treat systemic or local *Candida spp*. infections. In healthy men, a mean maximum serum concentration Cmax = 9.1–11 µM was achieved after a loading dose of 70 mg caspofungin^10^.

In order to successfully treat mycotic infections in different organs, caspofungin has to reach therapeutic concentrations in many tissues or regions including the liver and the lower airways of the lungs that exceed plasma concentrations^11,12^. This distribution pattern depends on the physiology of specific organs^13^. In critically ill patients and in perfused isolated rat hearts, we have previously reported that caspofungin has severe effects on cardio-circulatory function^14–16^. Caused by impaired contractility of cardiomyocytes as a result of changes in [Ca^2+^]_i_ levels^17^. It is therefore of interest to determine whether caspofungin may also interact with the homeostasis of [Ca^2+^]_i_ in airway epithelial cells. [Ca^2+^]_i_ is part of many cellular signal cascades that are a precondition to the altering of cell functions such as the beat rate of cilia. In order to find out, we investigated whether caspofungin also changes the [Ca^2+^]_i_ in isolated human tracheal epithelial cells (HTEpC) and tried to elucidate the underlying regulatory mechanism.

We measured [Ca^2+^]_i_ using the fluoroprobe FURA-2 AM. To determine the amount of Ca^2+^ ions in the ER, we used Mag-Fluo-4 AM. Pharmacological approaches were used to identify signal pathways that are involved in the regulation of [Ca^2+^]_i_. The data gathered by this study should expand our knowledge of the interactions of this antifungal drug in terms of the regulation of [Ca^2+^]_i_ in airway epithelial cells, which can be considered to be part of the immune system.

## Materials and methods

### Calcium imaging in isolated epithelial cells

For calcium imaging, we used the human tracheal epithelial cell line (HTEpC, C12644, PromoCell, Heidelberg, Germany). Cells were cultivated in an Airway Epithelial Cell Growth Medium Kit (C-21160) containing the Airway Epithelial Cell Growth Medium Supplement Pack (C-39160, PromoCell, Heidelberg, Germany). The airway epithelial cells were kept in a humidified chamber at 37 °C with air containing 5% CO_2_. For the Ca^2+^ imaging, cells were seeded onto laminin-coated coverslips. Dye loading with 2.5 µM FURA-2 AM (Biotium, Fremont, CA, USA) in darkness was performed in a 4-(2-hydroxyethyl)-1-piperazineethanesulfonic acid (HEPES) buffer for 30 min at 37 °C. For the contents of the HEPES buffer, see the section on drugs and buffer solutions. After the loading period, the cells were rinsed in fresh HEPES buffer and were transferred to the recording chamber of an upright fluorescence microscope equipped with 20 × immersion lenses (BX50 WI, Olympus, Hamburg, Germany), which contains 2 ml HEPES. The excitation light was provided by a 50 W xenon lamp. The microscope was equipped with a dichromatic excitation longpass mirror (400 nm).

The ratiometric dye, FURA-2 AM, was excited at 340 nm and 380 nm (± 8 nm) when equipped with bandpass excitation filters. The emitted fluorescence was directed through a dichromatic shortpass filter of 560 nm to a bandpass filter of 510 nm.

### Imaging of luminal Ca^2+^ ions in the ER of tracheal epithelial cells

For calcium imaging in the ER, HTE cells were seeded on laminin-coated coverslips. To assess the intraluminal Ca^2+^ concentration ([Ca^2+^]_ER_) within the ER, the cells were loaded with 5 µM of the low-affinity calcium indicator Mag-Fluo-4 AM (Kd = 22 µM, Invitrogen, Paisley, UK) in darkness at 37 °C for 60 min. Afterward, the cells were rinsed in HEPES buffer for 20 min and were then transferred into the recording chamber of the fluorescence microscope equipped with 20 × immersion lenses (BX50 WI, Olympus, Hamburg, Germany), which also contains 2 ml HEPES. Experiments were performed in both Ca^2+^-containing and Ca^2+^-free HEPES buffer (for contents see below). As single wavelength indicators are difficult to calibrate, we used normalized Mag-Fluo-4 signals (F/F_o_) to express changes in the luminal [Ca^2+^]_ER_. Pharmacological agents were applied with a blunt pipette into the recording chamber. The Mag-Fluo-4 was then excited at 490 nm (± 10 nm) and the emitted fluorescence was directed through a dichromatic shortpass filter of 560 nm to a bandpass filter of 510 nm.

### Fluorometric ROS measurements in tracheal epithelial cells under exposure to caspofungin

As an indicator of reactive oxygen species (ROS), we measured hydrogen peroxide (H_2_O_2_) in collected cell culture supernatants by using the fluorometric Hydrogen Peroxide Assay Kit (PromoCell, Heidelberg, DE). HTE cells were seeded on laminin-coated coverslips and were incubated with 60, 90 or 120 µM caspofungin (CAS) in 1 ml HEPES solution for 30, 60 and 90 min. Additionally, we incubated cells without adding CAS as a control. The cell supernatant (250 µl) was collected and centrifuged at 1,200 G for 15 min to remove all cell particles. To remove the proteins, the sample was then filtered through a 10 kDa MW spin filter (CAT.No. PK-CA577-1997, PromoCell, Heidelberg, DE). Samples were subsequently prepared in duplicate as follows. 50 µl of the samples were mixed with 50 µl Reaction Mix (Horse Radish Peroxidase, OxiRed Probe solution, Assay Buffer) in a 96-well plate (Costar, Corning, NY, USA) and were incubated for 10 min. A H_2_O_2_ standard was also established in duplicate with 0, 0.1, 0.2, 0.3, 0.4, or 0.5 nm/well. Fluorescence was measured (Ex/Em = 535/587 nm) in a microplate reader (Tecan, Maennedorf, CH). To calculate the H_2_O_2_ quantity, a H_2_O_2_ standard curve was plotted after background subtraction using the equation:

$${\text{C}} = {\text{Sa}}/{\hbox{Sv}}$$

where Sa = sample amount [pmol], and Sv = sample volume [µl]).

### Drugs and buffer solutions

The following drugs were administered: caspofungin (60 µM or 120 µM diluted in 20 µl H_2_O, MSD, Kenilworth, USA), NiCl_2_ (50 µM, Roth, Karlsruhe, Germany). Caffeine (30 mM, Roth, Karlsruhe, Germany) was sufficient to completely deplete ER Ca^2+^ stores in these cells. 2,4-dinitrophenol (DNP, 25 µM, Sigma-Aldrich, St. Louis, USA) was used to deplete mitochondrial Ca^2+^ stores by depolarization of mitochondrial membrane potential, 2-Aminoethoxydiphenylborane (2-APB, 40 µM, TOCRIS Bioscience, Bristol, UK); a high ryanodine concentration (40 µM, TOCRIS Bioscience, Bristol, UK) was used to inhibit Ca^2+^ liberation from ER Ca^2+^ stores. Ryanodine, 2-APB, and DNP were diluted in Dimethylsulfoxide (DMSO), and end concentrations were achieved by applying the stock solution directly to the buffer solution in the recording chamber. The DMSO did not exceed volumes of 20 µl in 2 ml HEPES in order to prevent the induction of unspecific disturbances of the cilia-bearing cells.

All preparations and experiments were carried out in HEPES solution consisting of 20 mM HEPES, 4.5 mM KCl, 2.5 mM CaCl_2_, 11 mM glucose, 140 mM NaCl, and 1 mM MgCl_2_. The pH was adjusted to 7.4 using 4 M NaOH at 30 °C, or 4–8 °C for tissue preparation. For Ca^2+^-free solutions, CaCl_2_ was replaced by 1 mM ethylene glycol tetra-acetic acid (EGTA). A vitality test was performed at the end of each recording with KCl solutions (200 mM). Solutions containing 200 mM KCl also contained 2.5 mM CaCl_2_.

### Statistical analysis

A Mann–Whitney U test was used to compare equivalent measuring points from different experiments, and a Wilcoxon rank-sum test was used to compare dependent variables. Statistical data evaluation and testing were performed using the GraphPad PRISM (Version 5.04) software (GraphPad Software, La Jolla, CA, USA).

## Results

### Changes in [Ca^2+^]_i_ in tracheal epithelial cells

**Figure 1:**
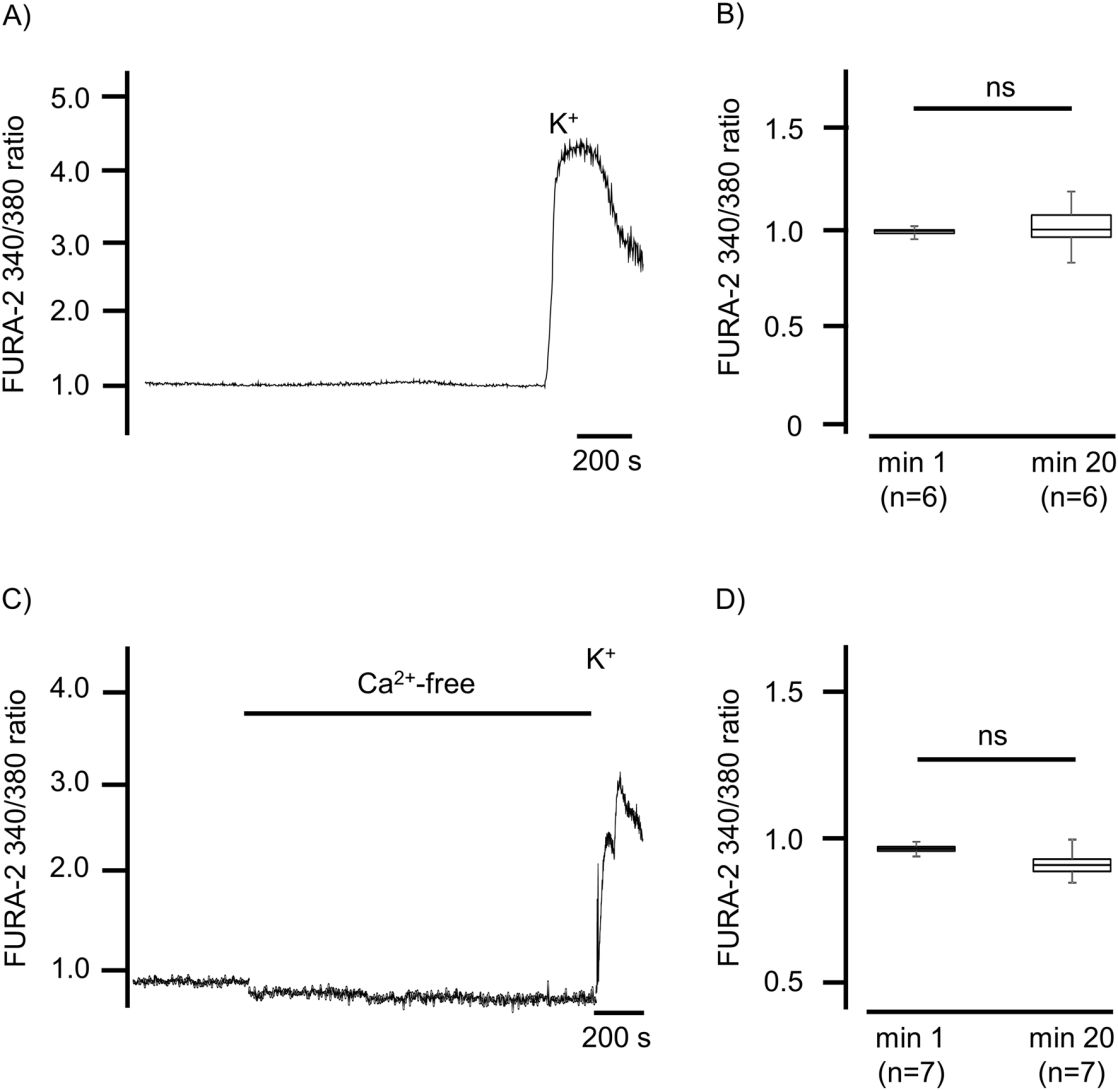
Prolonged incubation of tracheal epithelial cells in buffer solutions did not change [Ca^2+^]_i_. (**A**) The [Ca^2+^]_i_ was indicated by a FURA-2 ratio of 340 nm/380 nm. As a control, isolated tracheal epithelial cells were kept under long-term incubation in Ca^2+^-containing 2.5 mM buffer. During this prolonged incubation period, the basal [Ca^2+^]_i_ did not change. The vitality test at the end of each experiment was carried out by applying KCl (200 mM), inducing a rapid Ca^2+^-influx and an increase in [Ca^2+^]_i_. (**B**) The initial levels of [Ca^2+^]_i_ had not changed when they were briefly measured at the end of the exposure period before the application of KCl. (**C**) The prolonged incubation of tracheal epithelial cells in Ca^2+^-free buffer solution did not change the [Ca^2+^]_i_. The application of KCl (200 mM, containing 2.5 mM CaCl_2_) precipitated an increase in [Ca^2+^]_i_. (**D**) In the Ca^2+^-free buffer solution, the initial [Ca^2+^]_i_ also did not change when it was briefly measured at the end of the exposure period before the KCl application (n = number of individual experiments, ns = not significant, Wilcoxon rank-sum test).

The [Ca^2+^]_i_ was expressed as changes in the FURA-2-fluorescence ratio. For the controls, isolated tracheal epithelial cells were rested in Ca^2+^-containing or in Ca^2+^-free buffer solutions for more than 800 s. In the cells resting in Ca^2+^-containing buffer, the FURA-2-fluorescence ratio did not change significantly during this period (1.09 ± 0.08, p = 0.12, compared to initial FURA-2-fluorescence ratio). At the end of all the experiments, we applied a brief pulse of KCl (200 mM), and only the cells that responded to this vitality test by showing a significant increase in the FURA-2-fluorescence ratio were included for further statistical evaluation (Fig. 1). Cells resting in the Ca^2+^-free buffer medium also showed no changes in the FURA-2-fluorescence ratio throughout the entire exposure period (0.89 ± 0.03, p = 0.09, Wilcoxon rank-sum test, Fig. 1C, D). However, these cells also responded to a brief KCl pulse with an increase in the FURA-2-fluorescence ratio.

**Figure 2:**
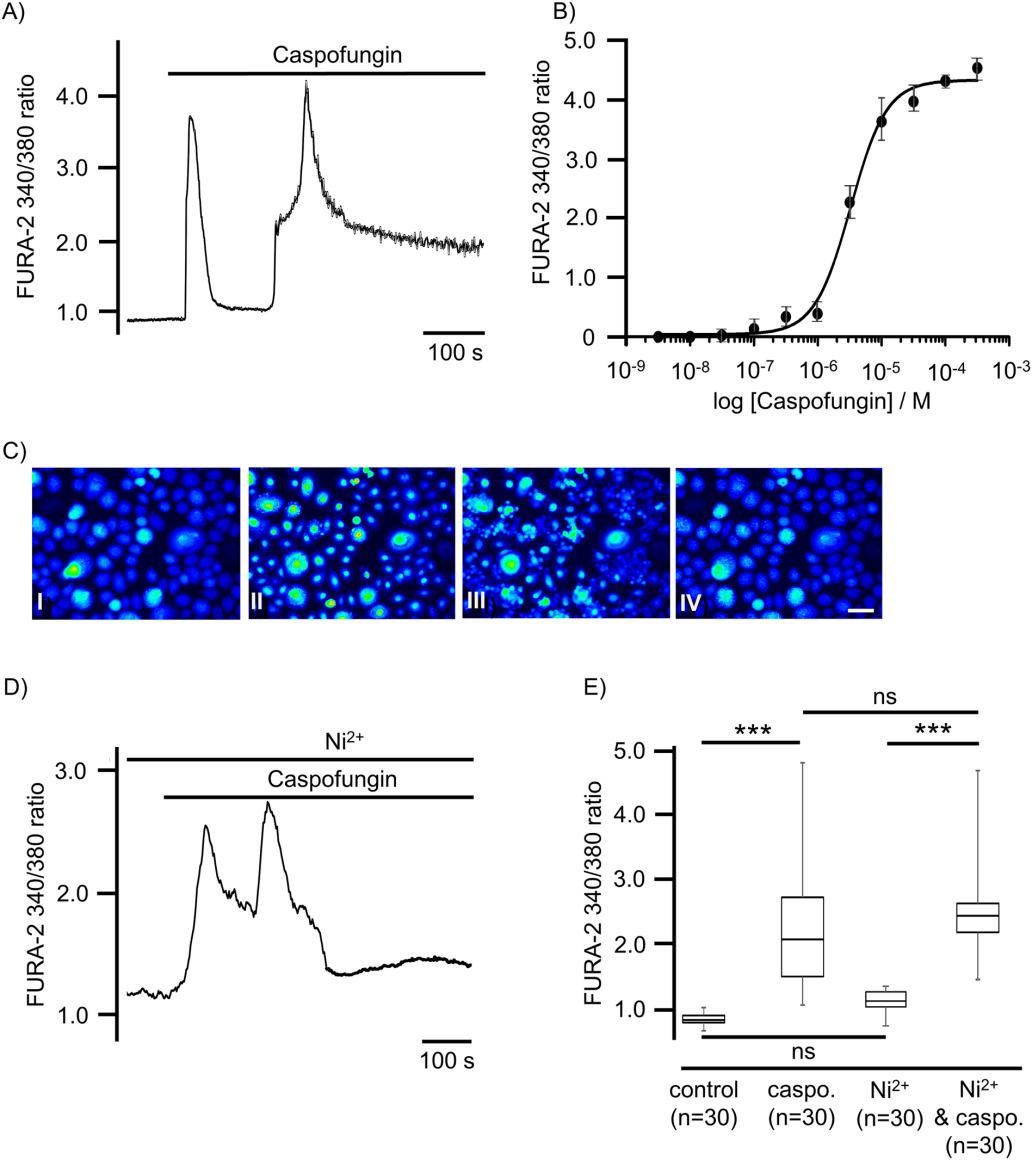
In tracheal epithelial cells, caspofungin triggers an increase in [Ca^2+^]_i_. (**A**) In the Ca^2+^-containing buffer solution (2.5 mM), the application of caspofungin (60 µM) induced a sudden rise in the FURA-2 ratio displaying [Ca^2+^]_i_. In the majority of the investigated cells (n = 21) we observed two transient prolonged peaks in [Ca^2+^]_i_, whereas the remaining cells (n = 4) showed a single transient rise of [Ca^2+^]_i_. (**B**) The amplitude of the initial Ca^2+^ increase induced by the caspofungin in the tracheal epithelial cells is concentration-dependent, with an EC_50_ of 55 µM. The dose–response curve fits a Hill equation. (**C**) Fluorescence images of the FURA-2 ratio converted into false colors that were taken at different time points from a prolonged recording of caspofungin application. I: Early response of cells to caspofungin application (50 s after caspofungin application); II: Further response of cells to caspofungin application with an increase in FURA-2 ratio (100 s after caspofungin application); III: Prolonged elevation of FURA-2 ratio (200 s after caspofungin application); IV: Prolonged elevated FURA-2 ratio (approx. 600 s after caspofungin application and just before KCl administration). (**D**) Ni^2^^+^ was applied in order to inhibit the influx of Ca^2+^ ions into the tracheal epithelial cells. The subsequent application of caspofungin still evoked a transient rise in [Ca^2+^]_i_ that was independent of the Ca^2+^ influx. (**E**) A significant increase in [Ca^2+^]_i_ was triggered when exposed to caspofungin (60 µM). Inhibition of membrane-bound Ca^2+^ channels by application of Ni^2^^+^ ions did not reduce the amplitudes of a transient rise in [Ca^2+^]_i_ (scale bar in **C:** 20 µm. n = number of individual cells, ns = not significant, ***p < 0.001, Mann–Whitney U test).

When the tracheal epithelial cells were exposed to caspofungin (60 µM) in Ca^2+^-containing buffer, almost all of the investigated cells immediately showed an elevation in [Ca^2+^]_i_. We observed a majority of cells responding with two subsequent Ca^2+^-transients with a maximum of 2.46 ± 0.29 (FURA-2-fluorescence ratio, n = 30). The first rapid increasing Ca^2+^ transient returned to the baseline, while the second one showed a rapid increase in [Ca^2+^]_i_ which did not return to the baseline during the remaining exposure period (Fig. 2A). Increasing the caspofungin concentration to 120 µM resulted in prolonged elevation of [Ca^2+^]_i_ that remained above the baseline at the end of the exposure period. There was a caspofungin dose-dependency of maximum amplitudes of initial Ca^2+^ transients (Fig. 2B). The application of NiCl_2_ was used to inhibit Ca^2+^ entry via the plasma-membrane-bound Ca^2+^ channels. However, it did not prevent the peak rise in [Ca^2+^]_i_ induced by caspofungin (60 µM, 2.47 ± 0.18, p = 0.093, n = 30, Fig. 2B, E).

### Caspofungin induces a rise in [Ca^2+^]_i_ independent of external Ca^2+^ ions

**Figure 3:**
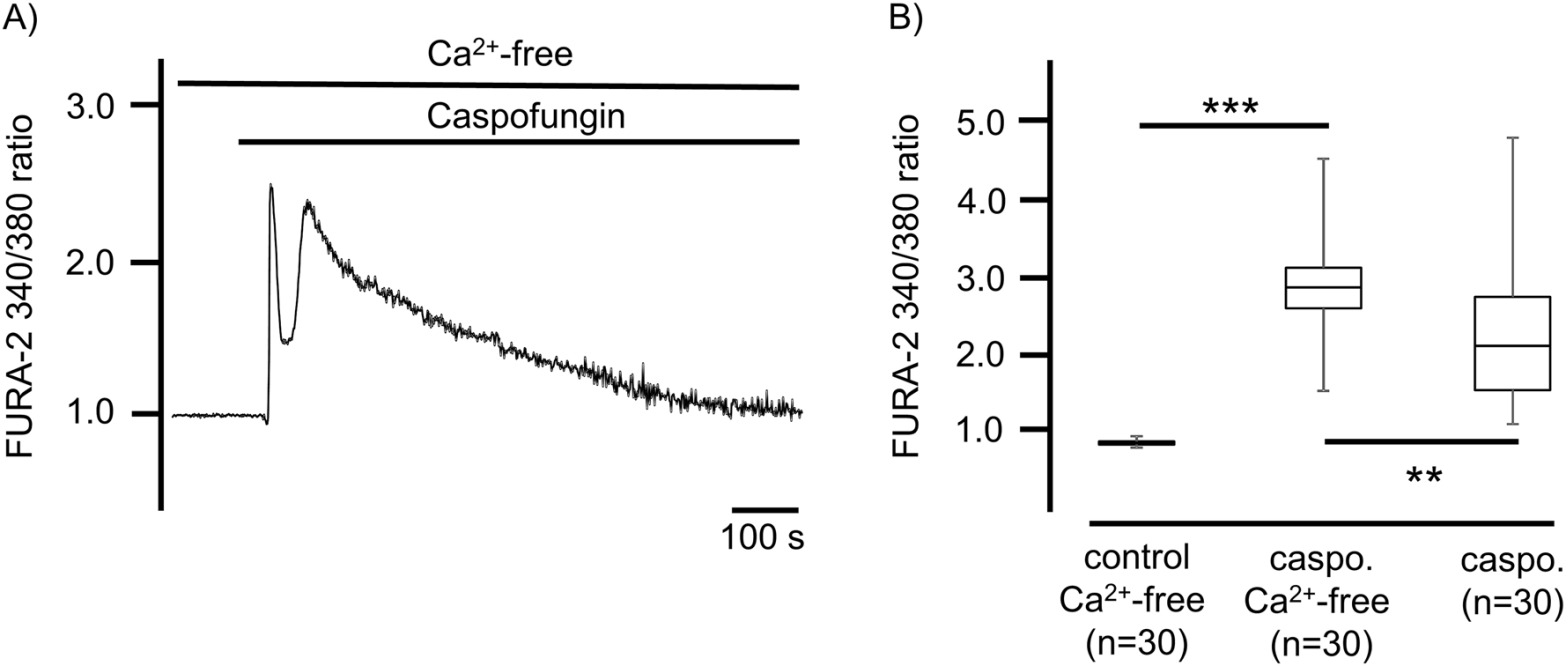
The caspofungin-induced rise in [Ca^2+^]_i_ is independent of external Ca^2+^ ions. (**A**) In the tracheal epithelial cells kept in Ca^2+^-free buffer solution, the application of caspofungin induced two consecutive elevations in [Ca^2+^]_i_. The second rise slowly decreased over time but remained above the baseline at the end of the exposure period. (**B**) The application of caspofungin in the Ca^2+^-free buffer medium induced a significant increase in [Ca^2+^]_i_. This rise in [Ca^2+^]_i_ was also significantly higher than the rise triggered by caspofungin in Ca^2+^-containing buffer solution (n = number of individual cells, ns = not significant. The horizontal bars in the experimental recordings depict the exposure periods to the Ca^2+^-free buffer solution and caspofungin application ,**p<0.01. ***p < 0.001, Mann–Whitney U test).

To further elucidate the caspofungin-induced rise in [Ca^2+^]_i_, we applied caspofungin to tracheal epithelial cells in Ca^2+^-free solutions. Exposure to caspofungin in the Ca^2+^-free solution evoked a rise in [Ca^2+^]_i_ in all of the investigated cells (n = 30) which was biphasic, and which remained above the baseline level until the end of the experimental observation period (Fig. 3A). The peak rise in [Ca^2+^]_i_ was significantly higher in the Ca^2+^-free buffer solutions (2.95 ± 0.72, n = 30) than the peak transients observed in the Ca^2+^-containing buffer solutions (2.27 ± 0.17, n = 30, p < 0.01; Fig. 3B).

### Caspofungin liberates Ca^2+^ from internal stores

In order to determine the source of the Ca^2+^ ions that increase the levels of [Ca^2+^]_i_ during caspofungin exposure, we pharmacologically depleted the known intracellular Ca^2+^ stores, e.g., DNP (25 µM) was used in Ca^2+^-free buffer solutions to deplete the mitochondrial Ca^2+^ stores. This uncoupling of the respiratory chain induces a complete depolarization of the mitochondrial membrane potential eliciting an efflux of Ca^2+^ ions from these organelles.

**Figure 4:**
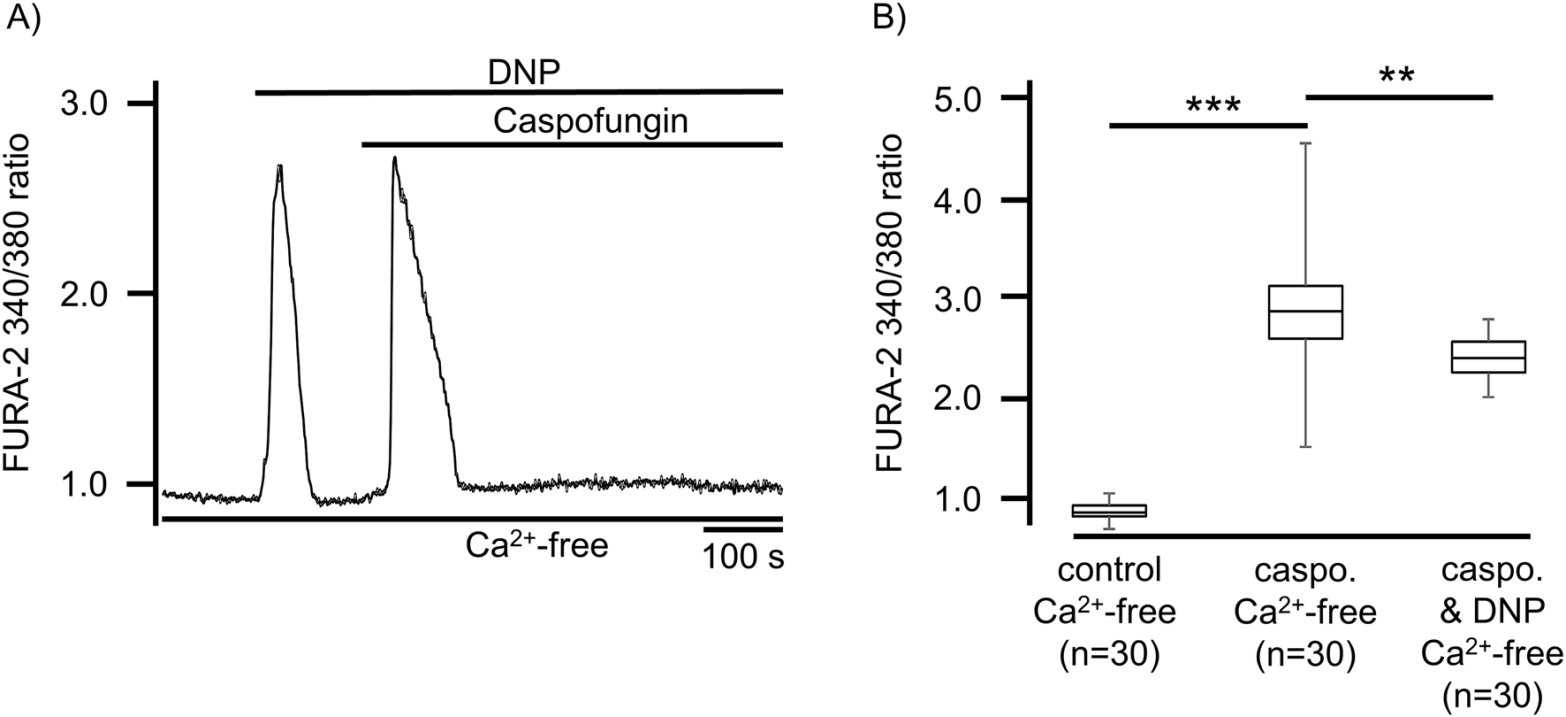
Caspofungin does not liberate Ca^2+^ ions from mitochondrial Ca^2+^ stores. (**A**) In a Ca^2+^-free buffer solution, mitochondrial Ca^2+^ stores were depleted by DNP (25 µM). DNP application resulted in a transient increase in [Ca^2+^]_i_ before returning to the baseline level. Nevertheless, when in the presence of DNP, caspofungin induced a rapid transient rise in [Ca^2+^]_i_ without a prolonged elevation. (**B**) The amplitude of the rise in [Ca^2+^]_i_ induced by caspofungin in the presence of DNP was lower than the elevation of [Ca^2+^]_i_ triggered by caspofungin without any depletion of mitochondrial Ca^2+^ stores (the horizontal bars in the experimental recordings depict the exposure periods of defined pharmacological agents. n = number of individual investigated cells, **p < 0.01, ***p < 0.001, Mann–Whitney U test).

The Ca^2+^ efflux from the mitochondria was visible by brief Ca^2+^ transients (increase in FURA-2 ratio) that almost immediately returned to baseline levels (Fig. 4A). The following exposure to caspofungin (60 µM), still in the presence of DNP, evoked transient elevation in [Ca^2+^]_i_ that always returned to the baseline. The transient increases in Ca^2+^ ions, when exposed to caspofungin in the presence of DNP, were significantly less than the transient increases in Ca^2+^ ions when the mitochondrial stores were not depleted (2.41 ± 0.07, n = 11, p < 0.01, Fig. 4B).

**Figure 5:**
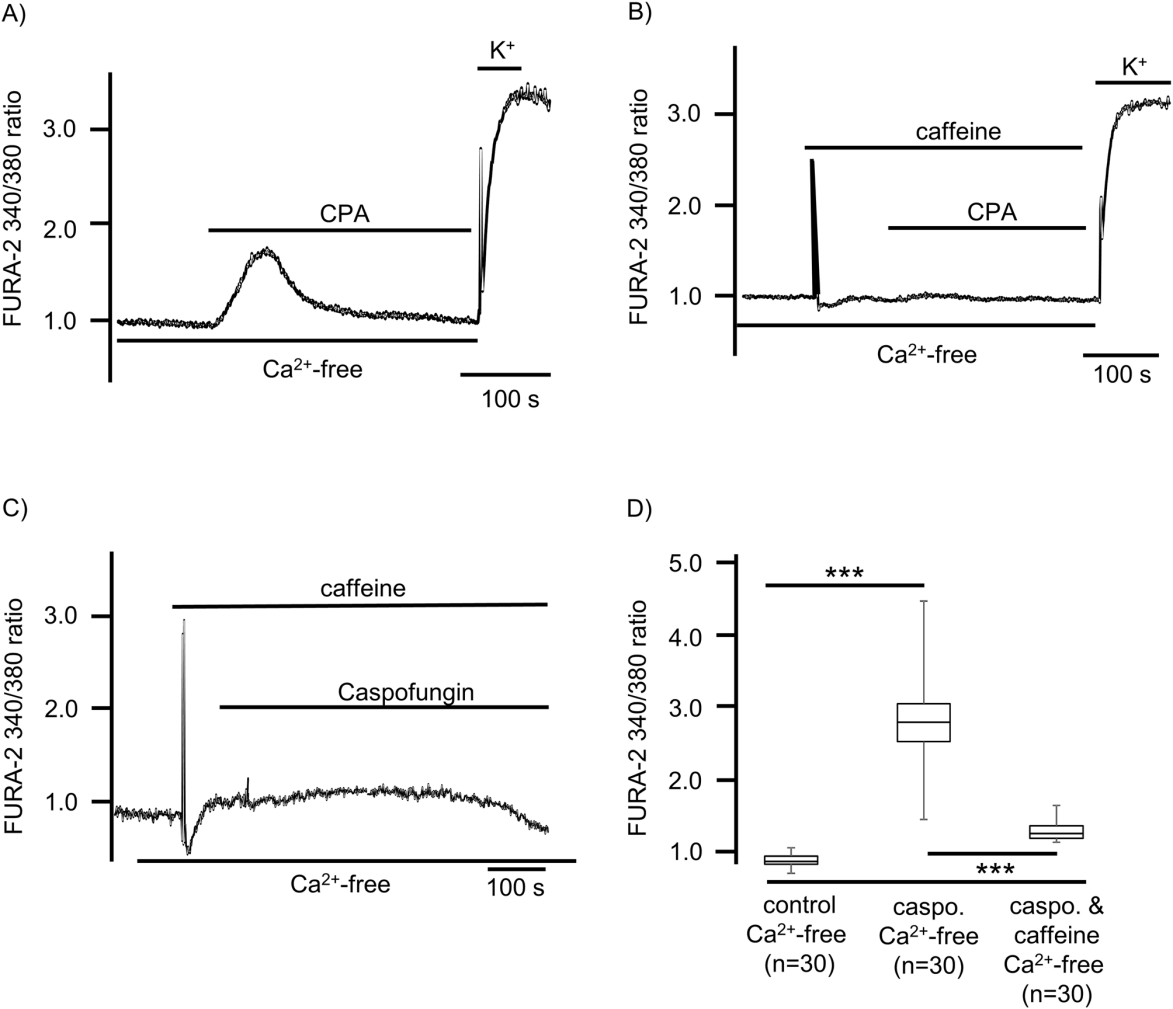
ER Ca^2+^ stores contribute to caspofungin-induced increases in [Ca^2+^]_i_. (**A**) The ER Ca^2+^ stores are identified by their sensitivity to the SERCA inhibitor CPA (10 µM). Exposure to CPA led to a slow increase in [Ca^2+^]_i_ that returned to baseline levels while still in the presence of CPA. (**B**) Caffeine (30 mM) also depleted the ER Ca^2+^ stores leading to a rapid increase in [Ca^2+^]_i_ that soon returned to baseline levels. The subsequent application of CPA did not lead to a further rise in [Ca^2+^]_i_, demonstrating that the caffeine had already depleted ER Ca^2+^ stores that are identical to CPA-sensitive Ca^2+^ stores. (**C**) Applying 30 mM of caffeine was enough to deplete the ER Ca^2+^ stores. The consecutive application of caspofungin while in the presence of caffeine did not change the [Ca^2+^]_i_. Under these conditions, no Ca^2+^ transients were observed. (**D**) The [Ca^2+^]_i_ measured under caffeine application and subsequent caspofungin application was significantly lower than the rise in [Ca^2+^]_i_ measured when the ER’s Ca^2+^ stores were not depleted (the horizontal bars in the experimental recordings depict the exposure periods of defined pharmacological agents. n = number of individual investigated cells, **p < 0.01, ***p < 0.001, Mann–Whitney U test).

Ca^2+^ stores in the ER were identified and depleted by cyclopiazonic acid (CPA, 10 µM), a reversible inhibitor of SERCA. In a Ca^2+^-free buffer solution, CPA induced a prolonged increase in [Ca^2+^]_i_ that slowly returned to baseline levels (Fig. 5A). Cell vitality was verified by exposure to a depolarizing concentration of KCl (200 mM, Fig. 5A). The depletion of ER Ca^2+^-stores by caffeine (30 mM) led to a rapid but transient increase in [Ca^2+^]_i_. The subsequent application of CPA (10 µM) had no further effect, demonstrating that caffeine had already depleted the ER Ca^2+^ stores, which are identical to CPA-sensitive stores (Fig. 5B).

In further experiments, exposing the cells to caffeine led to a brief Ca^2+^ transients of the FURA-2 fluorescence ratio that almost immediately returned to the baseline. The subsequent application of caspofungin (60 µM or 120 µM) had no effect on any tracheal epithelial cells investigated.

We observed no increase in [Ca^2+^]_i_ in the presence of caspofungin (1.32 ± 0.02, n = 30, p = 0.1 when compared to the FURA-2 fluorescence ratio after the addition of caffeine but before caspofungin application). However, Ca^2+^ transients were significantly reduced vs. Ca^2+^ transients induced by caspofungin in Ca^2+^-free buffer solutions (p < 0.001, Fig. 5C, D.

### Caspofungin enhances [Ca^2+^]_i_ via the activation of IP3 and ryanodine receptors

**Figure 6:**
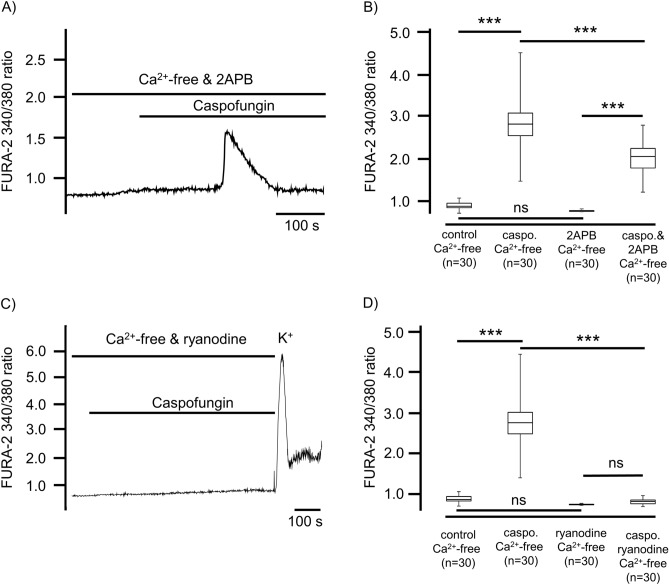
The caspofungin-induced rise in [Ca^2+^]_i_ depends on ryanodine and IP_3_ receptor activity. (**A**) In the Ca^2+^-free buffer solution, IP_3_ receptors were inhibited using 2-APB (40 µM), which did not affect the baseline [Ca^2+^]_i_. The application of caspofungin evoked a transient rise in [Ca^2+^]_i_ above the baseline. (**B**) The amplitude of the transient rise in [Ca^2+^]_i_ induced by caspofungin was significantly lower than the rise in [Ca^2+^]_i_ measured without the application of 2-APB (Mann–Whitney U test). The elevation in [Ca^2+^]_i_ induced by caspofungin in the presence of 2-APB was significantly higher than the baseline values (Wilcoxon rank-sum test). (**C**) The application of ryanodine (40 µM) did not change the basal [Ca^2+^]_I_ and the application of caspofungin did not lead to an increase in [Ca^2+^]_i_. The application of KCl at the end of the exposure period demonstrated the vitality of the cells. (**D**) The application of ryanodine did not change the baseline [Ca^2+^]_i_ levels in the Ca^2+^-free buffer solution. The application of caspofungin did not lead to an increase in [Ca^2+^]_i_. No Ca^2+^ transients were observed and the [Ca^2+^]_i_ was significantly lower than the rise in [Ca^2+^]_i_ measured after caspofungin application in Ca^2+^-free solution without inhibition of the ryanodine receptors (the horizontal bars in the experimental recordings depict the exposure periods of defined pharmacological agents. n = number of individual investigated cells, ns = not significant, **p < 0.01, ***p < 0.001, Mann–Whitney U test).

To evaluate the route by which Ca^2+^ ions are liberated by caspofungin from the ER’s Ca^2+^ stores, we pharmacologically inhibited the known Ca^2+^ pathways. 2-APB (40 µM) was applied to Ca^2+^-free extracellular solutions in order to inhibit inositol-1,4,5-triphosphate (IP_3_) receptors prior to caspofungin application. 2-APB had no effect on the FURA-2 fluorescence ratio indicating transients of [Ca^2+^]_i_ (1.04 ± 0.003, n = 30, p = 0.21, Fig. 6A, B). The subsequent application of caspofungin still induced single Ca^2+^ transients with a significantly reduced peak to 2.04 ± 0.08 (n = 30, p < 0.001) compared to Ca^2+^ transients induced by caspofungin alone (2.95 ± 0.72, Fig. 6B). However, we observed no prolonged elevation in the FURA-2 fluorescence ratio, which we observed when the Ca^2+^-free buffer solution was exposed to caspofungin (Fig. 3A). In a further series of experiments, we inhibited the ryanodine receptors using 40 µM ryanodine. This concentration of ryanodine did not induce an efflux of Ca^2+^ from ER stores, as demonstrated by the unaltered FURA-2 fluorescence ratio (Fig. 6C, D).

The application of caspofungin in the presence of ryanodine did not evoke any increase in the FURA-2 fluorescence ratio, and no Ca^2+^ transients in any of the investigated cells were observed (Fig. 6C, D). The [Ca^2+^]_i_ was no different to the values obtained prior to caspofungin application (1.1 ± 0.01, n = 30, p = 0.52). At the end of each experiment, Ca^2+^ transients were still elicited by applying KCl, demonstrating the vitality of the cells (Fig. 6C).

### Caspofungin depletes ER Ca^2+^ stores in tracheal epithelial cells

**Figure 7:**
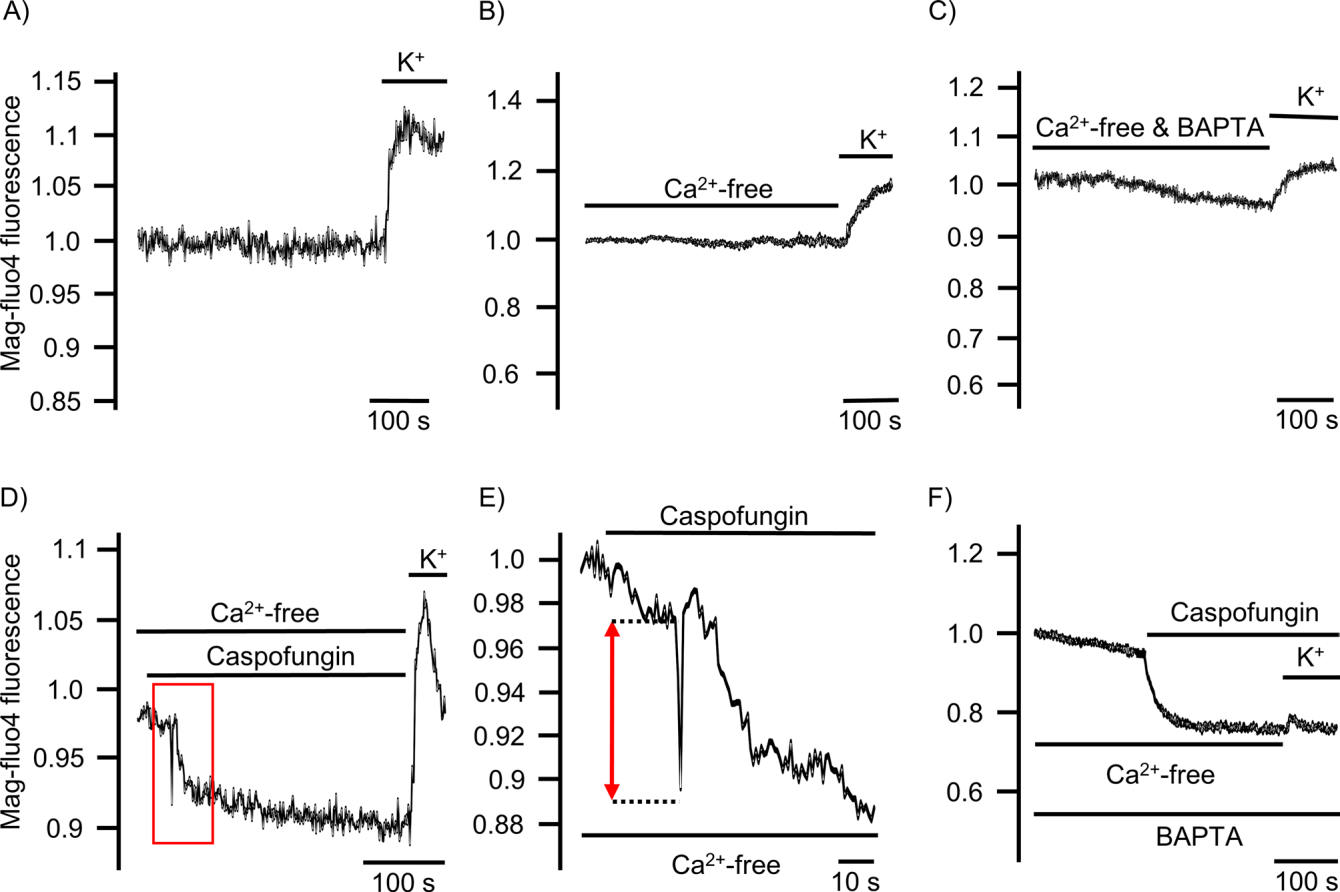
Caspofungin depletes ER Ca^2+^ stores. (**A**) Tracheal epithelial cells resting in Ca^2+^-containing buffer showed no changes in Mag-Fluo-4 fluorescence. (**B**) Ca^2+^-free buffer solution did not change the Mag-Fluo-4 fluorescence after prolonged exposure. (**C**) Cells were loaded with BAPTA to buffer cytosolic free Ca^2+^ ions in order to reduce [Ca^2+^]_i_. These resting cells showed only minor changes in the Mag-Fluo-4 signal, assuming that the ER Ca^2+^ stores remained constantly filled. (**D**) Exposure to caspofungin led to a rapid decrease in Mag-Fluo-4-fluorescence (red box) that almost immediately recovered back to baseline levels. Prolonged exposure to caspofungin then led to a slow decline in Mag-Fluo-4-fluorescence to a stable lower plateau. (**E**) Enlarged section of the initial spike from (**D**) (red box) showing the rapid kinetic and amplitude (red arrow) of the initial response to caspofungin exposure. (**F**) In cells exposed to caspofungin that were preloaded with BAPTA, the initial downward spike in Mag-Fluo-4-fluorescence completely disappeared and a prolonged reduction in Mag-Fluo-4-fluorescence was recorded. Replenishing the stores with Ca^2+^ ions by depolarizing the cells using KCl had little effect because the BAPTA instantly buffered the cytosolic Ca^2+^ ions. The luminal ER Mag-Fluo-4-fluorescence remained low (the horizontal bars in the experimental recordings show the exposure periods of the defined pharmacological agents).

**Figure 8:**
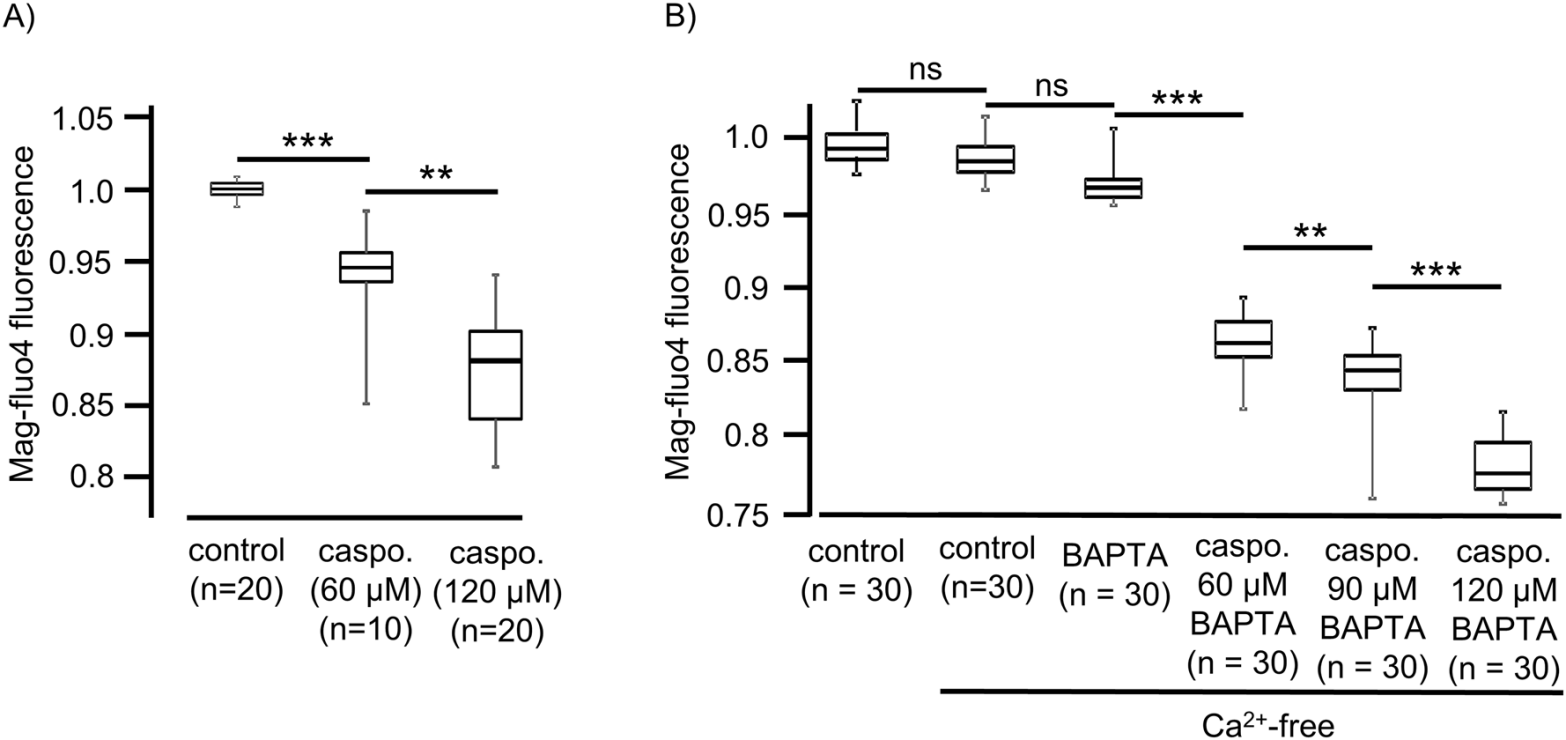
The effects of caspofungin effects on ER Ca^2+^ stores are concentration-dependent. (**A**) Caspofungin induced an immediate downward spike in Ca^2+^ ions. The amplitudes of these transient Ca^2+^ spikes were concentration-dependent. Low caspofungin concentrations (60 µM) already induced the Ca^2+^ transients that were significantly augmented by high caspofungin concentrations (120 µM). (**B**) Prolonged depletion of ER Ca^2+^-stores was noted for different caspofungin concentrations in the presence of BAPTA. The sustained minimum signals of Mag-Fluo-4-fluorescence induced by prolonged exposure to caspofungin were concentration-dependent. The degree of ER Ca^2+^-store depletion was most pronounced under high caspofungin concentrations (120 µM), (n = number of individual investigated cells, ns = not significant, **p < 0.01, ***p < 0.001, two-way ANOVA).

For the controls, HTEpC-c cells loaded with Mag-Fluo-4 AM were rested in Ca^2+^-containing buffer (Fig. 7A), Ca^2+^ free HEPES buffer (Fig. 7B), and Ca^2+^-free HEPES buffer after prior loading with BAPTA-AM (Fig. 7C). None of these conditions reduced the Mag-Fluo-4 fluorescence signal displaying [Ca^2+^]_ER_, demonstrating that the ER Ca^2+^ stores were stable under resting conditions without significant leakage of Ca^2+^ ions into the cytosol (Fig. 7A). However, exposure to caspofungin rapidly altered the Mag-Fluo-4 fluorescence. In all the cells that were investigated, we observed a rapid decrease in fluorescence within a few seconds of exposure (Fig. 7D, E) that immediately recovered, before a slower and more prolonged decline in the fluorescence showed the [Ca^2+^]_ER_. The amplitude of the initial downward spike of Mag-Fluo-4 fluorescence and the degree of the prolonged reduction was concentration-dependent (Fig. 8A, B). A final exposure to the Ca^2+^-containing buffer solution with a depolarizing KCl concentration demonstrated a recovery of the Mag-Fluo-4 signal equivalent to the replenishment of Ca^2+^ stores within the ER (Fig. 7D).

The initial sharp decrease in Mag-Fluo-4 fluorescence completely disappeared when the cells were loaded with BAPTA prior to caspofungin exposure; only the slow, prolonged phase of the declining Mag-Fluo-4 was still visible. In addition, replenishing the ER Ca^2+^ stores by Ca^2+^-containing buffer and depolarizing the cells with KCl only had a limited effect, assuming that the cytosolic Ca^2+^ ions were instantly buffered by BAPTA (Fig. 7F).

**Figure 9:**
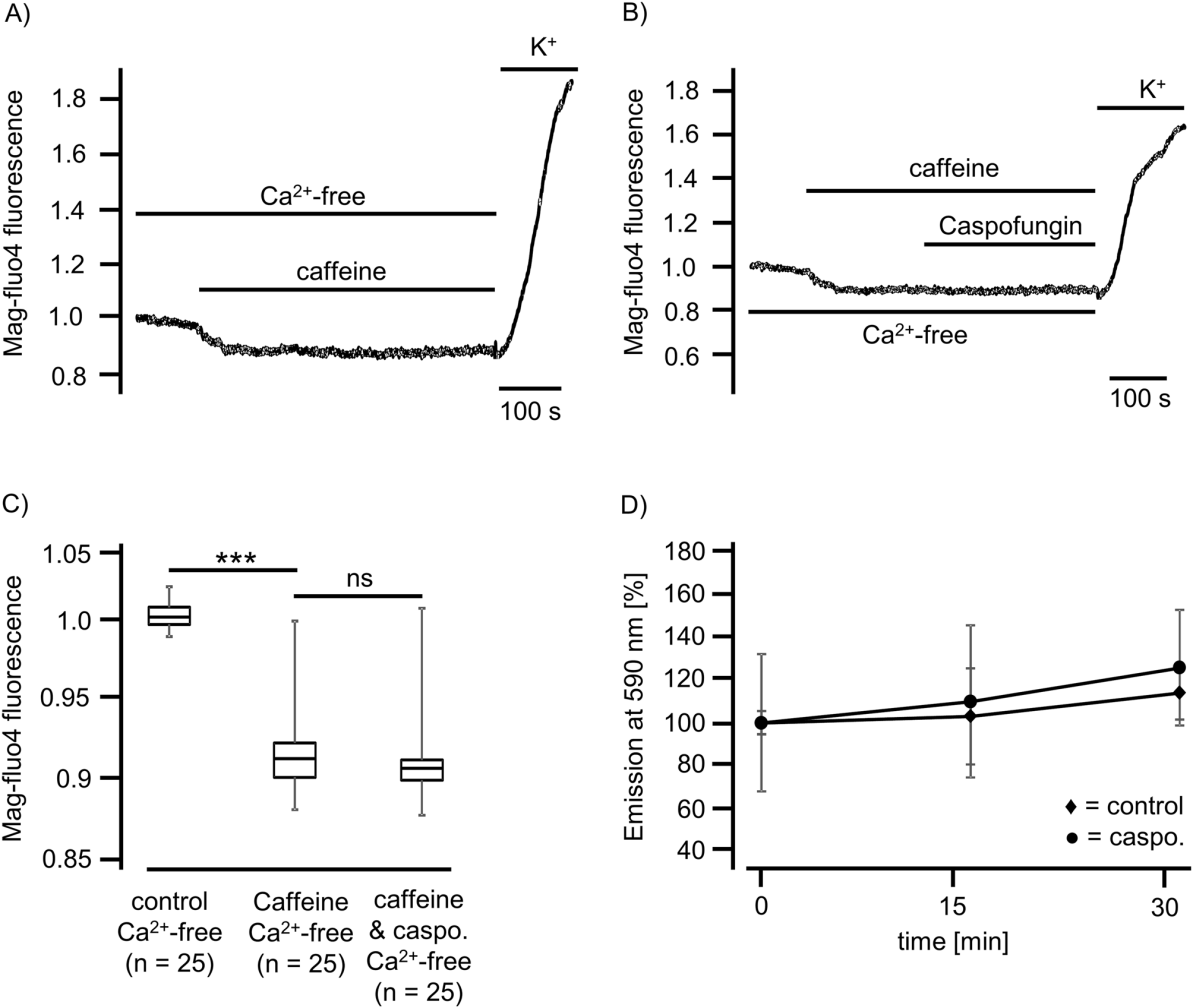
Caspofungin depletes caffeine-sensitive ER Ca^2+^ stores. (**A**) In the Ca^2+^-free buffer solution, exposure to caffeine (30 mM) led to a rapid and prolonged reduction in Mag-Fluo-4-fluorescence. The ER stores were replenished by a voltage-gated Ca^2+^ influx (exposure to 200 mM K^+^ ions). (**B**) Caffeine exposure induces the discharge of ER Ca^2+^ stores prior to caspofungin application. In addition, caspofungin did not induce a further reduction in Mag-Fluo-4-fluorescence, demonstrating that ER stores were already depleted by caffeine. (**C**) The addition of caffeine led to a significant reduction in the Mag-Fluo-4-fluorescence indicating that the Ca^2+^ stores in the ER were empty. Subsequent application of caspofungin in the presence of caffeine did not alter the Mag-Fluo-4-fluorescence any further. (**D**) Isolated mice tracheae were either exposed to caspofungin (120 µM) or kept as controls in buffer solutions. Under both conditions, we observed only minor alterations in ROS generation displayed by emission fluorescence at 590 nm. (the horizontal bars in the experimental recordings depict the exposure periods of defined pharmacological agents. n = number of individual investigated cells, ns = not significant, ***p < 0.001, two-way ANOVA).

The total depletion of ER Ca^2+^ stores was achieved by the application of caffeine (30 mM). The stores were replenished after subsequent exposure to a buffer solution containing depolarizing KCl (200 mM) and Ca^2+^ (2.5 mM), inducing a maximum rise in Mag-Fluo-4 fluorescence (Fig. 9A). In the presence of caffeine, exposure to caspofungin had no further effect. Neither was the initial downward Ca^2+^ spike visible nor was the prolonged reduction of Mag-Fluo-4 fluorescence further diminished (Fig. 9B, C).

### ROS generation under caspofungin exposure in the tracheal epithelium

Here, we were able to determine whether the observed effects of caspofungin on intracellular Ca^2+^ stores were caused by cellular ROS generation. In freshly isolated mice tracheae, we measured ROS generation under exposure to caspofungin (120 µM) using a fluorescence dye. The results showed no significant ROS generation in the control experiment or when the tracheae were exposed to caspofungin for 30 min (Fig. 9D).

## Discussion

In this study, which looked at isolated HTEpCs, we found evidence that caspofungin triggers a rise in [Ca^2+^]_i_ by releasing Ca^2+^ ions from caffeine-sensitive ER stores, primarily via a RyR pathway and via an IP_3_ regulated cascade to a lesser extent. This increase in [Ca^2+^]_i_ and the releasing of Ca^2+^ ions from the ER was slightly dependent on extracellular Ca^2+^ concentrations or transmembraneous Ca^2+^ influxes, even though we could not prove it by blocking transmembraneous Ca^2+^ channels by using NiCl2. It is possible that NiCl2 is not able to block all the transmembraneous Ca^2+^ channels of tracheal epithelial cells or that we used an insufficient concentration. However, the cells showed a reaction directly after the application of 500 µM NiCl2, implying that the dosage was sufficient. In most of the cells investigated, the rise in [Ca^2+^]_i_ was biphasic, whereby an initial transient rise in [Ca^2+^]_i_ was followed by a second prolonged rise in [Ca^2+^]_i_. This data is supported by our experiments using Mag-Fluo-4-fluorescence, which showed a rapid biphasic depletion of ER Ca^2+^ stores after exposure to caspofungin. These present findings are similar to the effect of acetylcholine on the tracheal ciliary beating rate, inducing a transient rise in [Ca^2+^]_i_ that is followed by a prolonged increase in the beating rate^8^. We found no evidence that caspofungin induces ROS generation since ROS are known to interfere with calcium-signaling pathways.

The stimulation of cilia-bearing cells in order to enhance their beating rate is the main task to rapidly convey heavy loads of particles and pathogens. This mechanism depends on the liberation of Ca^2+^ ions from internal stores^18^. It is also in line with reports that evidenced the activation of the ciliary beat frequency following the kinetics of [Ca^2+^]_i_^19^. We were also able to demonstrate that caspofungin activates intracellular signal transduction cascades that lead to the release of Ca^2+^ ions from ER stores via RyR. We also demonstrated that caspofungin depleted the Ca^2+^ stores in the ER.

However, although we still cannot conclude that caspofungin directly affects RyR, we have excluded any interaction with membrane-bound receptors or with their downstream reaction cascades. Therefore, with the present data, we can assume that caspofungin can penetrate tracheal epithelial cells and to activate specifically RyR. This is similar to cardiomyocytes, in which caspofungin also triggers the liberation of Ca^2+^ ions from the ER via RyR^17^.

### The permeability of caspofungin and its diffusion into mammalian tissues and cells

Caspofungin inhibits the synthesis of 1,3-β-D-glucan, which is an essential component of the fungal cell wall^20,21^. While caspofungin underlies very low excretion kinetics that result in high sustained plasma levels, it attains therapeutic concentrations in various tissues^22^. It is also characterized by its ability to diffuse into many organs and tissues in order to reach the concentrations necessary to treat invasive candidiasis; thus, its penetration into the central nervous system (CNS) is poor^23^. Even when the meninges are inflamed, the concentrations of caspofungin in the CNS are far below serum concentrations^24^. In contrast, caspofungin concentrations are highest in the liver, lungs, kidney, and spleen, where caspofungin can deploy its antifungal properties^13^. In the lungs, the concentration of caspofungin leads to favorable response rates of up to 62% in neutropenic patients suffering from pulmonary invasive fungal disease^25^.

In mammals, caspofungin mainly acts extracellularly against most *Candida* species. However, caspofungin, a membrane-anchored cyclic lipopeptide, is also able to penetrate mammalian cells and can reach high concentrations within the tracheal epithelium allowing it to act upon cilia-bearing cells. We can also conclude that caspofungin concentrations are also high in the epithelial lining fluid, and it may act from the luminal side onto epithelial cells.

Since caspofungin is used to treat pulmonary infections caused by *Candida spp.*, it is able to diffuse via the epithelium into the airway lumen so that it can reach these therapeutic concentrations. We found evidence that caspofungin can reach intracellular concentrations in isolated lower airway cells that are high enough to liberate Ca^2+^ ions from cell organelles. So far, the effects of caspofungin on intracellular organelles have only been described for a few cell types. In stimulated mammalian macrophages, caspofungin has been shown to reach concentrations that will have an effect on trapped *Candida glabrata*^26^. Recently, we described a change in the contractility of isolated cardiomyocytes via the liberation of Ca^2+^ ions from internal Ca^2+^ stores^15,17^. These data support our present findings and lead us to postulate that the diffusion of caspofungin into mammalian cells is not restricted to particular cell types; it seems rather a general characteristic of this antimycotic substance.

### The effects of caspofungin on intracellular Ca^2+^ stores

In our present study, we identified that caspofungin could trigger the release of Ca^2+^ ions from Ca^2+^ stores in the ER, mainly via a caffeine/ryanodine-sensitive pathway. We found no evidence for the liberation of Ca^2+^ ions stored in mitochondria, although these organelles do store Ca^2+^ ions under resting conditions (see Fig. 4). However, they primarily buffer Ca^2+^ ions when [Ca^2+^]_i_ exceeds a threshold of approximately 500 nM^9,27^. In liver cells and cardiomyocytes, caspofungin is known to disturb the electron transport within the mitochondrial respiratory chain by inhibiting complex I and III. The effect on complex III is probably caused by its interference with cytochrome C^28^. However, it is not known whether this mechanism also depolarizes mitochondrial membrane potential, which is essential for Ca^2+^ buffering in these organelles^9^. Here we measured no effect of caspofungin onto mitochondrial Ca^2+^ stores.

When the mitochondrial Ca^2+^ stores were depleted by DNP, exposure to caspofungin had little effect (see Fig. 4). This small decrease in caspofungin response can be explained by the inhibition of ATP synthesis due to the application of DNP application^29^. ATP usually powers the sarco-endoplasmic calcium ATPase (SERCA) that refills the ER’s Ca^2+^ stores. Therefore, the interrupted mitochondrial ATP synthesis after exposure to DNP reduces SERCA activity, which then leads to a slow Ca^2+^ leakage from the ER stores via different pathways^9,30^. Eventually, this results in a slightly reduced Ca^2+^ response during subsequent caspofungin application, which was what was observed here. Our observations provide evidence that the mitochondria in tracheal epithelial cells store Ca^2+^ ions under resting conditions but are not affected by caspofungin.

Under steady-state conditions, the influx of Ca^2+^ ions into the cytosol from the ER stores is balanced on the one hand by RyR, IP_3_, or alternative leaks that drive the efflux from the ER, and on the other hand by SERCA, which regulates Ca^2+^ influx into the ER resulting in a zero net flux of Ca^2+^ ions into the cytosol^31,32^. Ca^2+^ sparks are transient elevations of the [Ca^2+^]_i_ that are caused by a net efflux from the ER. This efflux is caused by an increased open probability of the RyR, which cannot immediately be offset by the SERCA-driven influx. Using caffeine to open the RyR pathway leads to a rapid and transient Ca^2+^ efflux that depletes the ER’s stores. The following balance of efflux and influx of Ca^2+^ ions to pre-caffeine levels while still in the presence of caffeine is caused by clearing the elevated [Ca^2+^]_i_ by pumping Ca^2+^ ions out of the cells or into alternative stores while the RyR is still activated and open^33^. The refilling of the ER stores cannot be achieved under these conditions, which is the reason why we observed no further Ca^2+^ transients when caspofungin was applied after the ER stores were depleted by the addition of caffeine. When caspofungin is applied to cells that have not had their ER stores depleted by caffeine, we observed Ca^2+^ transients in most of the cells.

In our experiments using Mag-Fluo-4 as an indicator for ER luminal Ca^2+^ contents, we observed a rapid decline in Mag-Fluo-4-fluorescence followed by a partial recovery, which eventually led to a prolonged decline in Mag-Fluo-4-fluorescence. The partial recovery is most probably a response to the SERCA balancing the efflux and influx of Ca^2+^ ions from the ER, as is generally assumed for many cell types^34^. A similar mechanism of Ca^2+^ depletion from the ER has been described previously for smooth muscle cells derived from different organs in rats^35,36^. Here, the prolonged decline of Mag-Fluo-4-fluorescence was caused by the continuous presence of caspofungin activating RyR.

Under these conditions, SERCA was unable to balance the influx and efflux of Ca^2+^ ions from the ER stores. Most probably, this net efflux is provoked by caspofungin triggering the increase in open state probability of RyR, since the kinetics of Ca^2+^ transients are similar. The clearance of cytosolic Ca^2+^ transients is caused by alternative clearance pathways, e.g., the transmembrane efflux, buffering, or by an uptake in alternative Ca^2+^ stores like mitochondria. We interpret these findings such that caspofungin specifically activates ER-bound RyR and depletes these Ca^2+^ stores. Caspofungin now provokes an imbalance in favor of a net Ca^2+^ efflux that results in an immediate increase in [Ca^2+^]_i_, which is either transient or sustained. The rapid amplitude in [Ca^2+^]_i_ under caspofungin exposure is caused by the steep function of Ca^2+^ content in the ER as has previously been described in cardiomyocytes^37,38^. This Ca^2+^ content contributes to the regulation of ciliary beat activity by the activation of different signal cascades^8^.

Alternatively, caspofungin may promote the dissociation of Ca^2+^ ions from cytosolic buffers as an alternative source for increasing [Ca^2+^]_i_. In many cell types, cytosolic Ca^2+^ is strongly buffered, and for every free cytosolic Ca^2+^ ion approximately 100 to 200 Ca^2+^ ions are bound to buffers^39,40^. In cardiomyocytes, the major buffers are troponin and SERCA, whereas in airway epithelial cells the Ca^2+^ is supposed to be buffered by calmodulin^8,34^. However, in this case, the kinetics of increased [Ca^2+^]_i_ would be different.

We have not observed biphasic Ca^2+^ transients or sustained elevations of [Ca^2+^]_i_. This is because Ca^2+^ pumps on the plasma membrane, the ER and mitochondrial Ca^2+^ uptake would still be fully active and would be sufficient to immediately reduce elevated [Ca^2+^]_i_ content.

Since the initial binding of Ca^2+^ ions to SERCA contributes significantly to Ca^2+^ buffering, it may be that caspofungin contributes to the dissocitaion of Ca^2+^ ions from these binding sites^41^. Nevertheless, in this case, we would observe Ca^2+^ transients while RyR was inhibited by ryanodine, or when the ER stores were depleted by caffeine. However, we did not observe these Ca^2+^ kinetics. These data argue against the assumption that caspofungin drives Ca^2+^ ions out of the buffering sites of the SERCA. Furthermore, the inhibition of SERCA by caspofungin would lead to a slow, sustained increase in [Ca^2+^]_i_ which then returns to the baseline, as we observed for CPA (Fig. 5)^9,30^. In contrast, we did not observe a slow increase in [Ca^2+^]_i_ under caspofungin exposure; the kinetic was always steep and rapid. In contrast, in many of the airway cells observed, the decay in the elevated [Ca^2+^]_i_ levels was slow. This prolonged kinetic may be caused either by reduced ATP content and subsequently reduced activity of ATP-driven ion pumps or by the inhibition of ion transporters. However, it is questionable whether caspofungin reduces ATP generation or inhibits ion transport and this needs to be investigated further.

In our experiments, we noted a reduced response to caspofungin while using 2-APB, which binds to the IP_3_ receptor. Since phospholipase-C activation and liberation of Ca^2+^ ions from Ca^2+^ stores via the IP_3_ receptor contribute to Ca^2+^ wave propagation in airway epithelia, we investigated whether caspofungin interferes with this signal cascade^42^. The reduced response to caspofungin exposure when 2-APB was applied may be caused by the activation of these receptors by caspofungin in competition with 2-APB. This assumption is supported by the fact that the response to caspofungin was delayed in the presence of 2-APB. Therefore, 2-APB might only be effective when caspofungin concentration is high enough to prevent 2-APB from binding. Further findings revealed that ryanodine abolished the calcium signals elicited by Caspofungin. Controversially, IP3-mediated calcium release seems not to occur in parallel to RyR-mediated calcium release. Nevertheless, we observed a reduced response to caspofungin in the presence of 2-APB. 2-APB is also known to modulate TRP channel, gap junctions, STIM ORAI channel conductance and I_CRAC_ in higher concentrations^43,44^. Owing to this lack of specificity for the IP_3_ receptor, 2-APB could also impair other calcium pathways and not particularly the activity of the IP_3_ receptor. On the other hand, our experiments were performed in calcium free buffer solution, so store-operated calcium entry (SOCE) could not occur. Further experiments are necessary to explore whether the IP3 receptor pathway is affected by using a more specific IP_3_ receptor blocker such as Xestospongin C or to examine SOCE pathways, which is more complicated due to diverse interactions and a finetuned orchestration of multiple targets. Regardless, we conclude that RyR is the principal target of caspofungin since we measured the main effects of caspofungin via this Ca^2+^ efflux pathway.

The observed sustained elevation of Ca^2+^ ions under caspofungin exposure may be due to several reasons, as a similar effect has been described when these cells are exposed to histamine or acetylcholine^8,45^. Most probably, this prolonged kinetic is caused by an imbalance between the influx and efflux of Ca^2+^ ions from the cytosol due to a slow clearance rate when reaching baseline levels of [Ca^2+^]_i_ during the observation period. These findings imply that caspofungin has effects on other cell organelles. It may also interfere with mitochondrial ATP synthesis, as has been described for mammalian mitochondria^28^. Under these circumstances, the remaining ATP supply from mitochondria, glycolysis or phosphocreatine stores is too low to fully activate Ca^2+^ ATPase in the plasma membrane. This mechanism prevents a rapid return of [Ca^2+^]_i_ to baseline levels. Beyond plasma membrane-bound, sodium-calcium exchangers are also activated when [Ca^2+^]_i_ is high and may blunt the peak of observed the Ca^2+^ transients^46^.

We also found no evidence that caspofungin depolarizes mitochondrial membrane potential, which is the driving force behind Ca^2+^ storage in these organelles. This is supported by our findings that the number of Ca^2+^ transients was only slightly altered when mitochondria were depolarized using DNP. Therefore, in many tracheal epithelial cells, caspofungin may have additional effects on other cellular structures that cause a prolonged elevation of [Ca^2+^]_i_, with the slow return to the baseline level due to an energetic restriction.

HTE cells, which are isolated cells from the human tracheal epithelium, are suitable for investigating the different effects and signal pathways under stable conditions, whereas freshly isolated human tracheal cells should only be used in further studies to underline these observations. Additionally, a contribution of alternative Ca^2+^ influx pathways, e.g. store operated calcium entry (SOCE) or IP_3_ receptors in the intact tracheal epithelium, should also be considered.

## Conclusion

Caspofungin liberates Ca^2+^ ions from internal ER stores and enhances [Ca^2+^]_i_ either transiently or in a sustained kinetic. This mechanism likely occurs due to the activation of a RyR pathway. A sustained elevation in [Ca^2+^]_i_ levels may be supported via energetic imbalances during caspofungin application. Further studies should focus on RyR activation by caspofungin, detecting its binding site to these receptors, and determining how cellular ATP synthesis is compromised by caspofungin.
